# The Ethics of Supporting Bereaved Parents Following Traffic Crash Fatalities

**DOI:** 10.21315/mjms2022.29.1.17

**Published:** 2022-02-23

**Authors:** Yusrita Zolkefli

**Affiliations:** Pengiran Anak Puteri Rashidah Sa’adatul Bolkiah (PAPRSB) Institute of Health Sciences, Universiti Brunei Darussalam, Brunei Darussalam

**Keywords:** ethics, bereavement, parents, incidents, healthcare professionals

## Abstract

We recognise that the death of a child is one of the most difficult events a family can experience. The article is based on a tragedy that occurred last year near Gunung Lang in Perak, Malaysia, in which a mother was the only survivor of a road accident in which she lost three young sons. It was reported that the husband, who was not involved in the crash, wanted to tell her about the loss. This raises two important moral questions: first, who would be the best person to break the news of the children’s death; second, what would be the healthcare professional’s ethical responsibility in advocating for and supporting the best interests of the bereaved parents? The case illustrates the need for further ethical reflection by healthcare professionals while supporting bereaved parents. Healthcare professionals must also be sensitive to their ethical responsibility to promote the best interests of grieving parents, which reflects higher vulnerability for bereaved parents.

Dear Editor,

I read with profound sadness the news article entitled ‘3 children killed in North-South Expressway (NSE) crash were siblings, mom severely injured’, published in December 2020 (1). In the horrific road accident near Gunung Lang in Perak, Malaysia, a mother (the sole survivor) sustained severe injuries and lost all three of her young sons. The news deeply moved me as we know that the death of a child is one of the most difficult events that can occur in a family. In reference to this, I would like to raise two important moral questions in the healthcare context: first, who would be the best person to break the news of the children’s death; and second, what would be the healthcare professional’s ethical responsibility in advocating for and supporting the best interests of the bereaved parents?

Firstly, in this case, the husband has made it clear that he will be the one breaking the news. However, is it ethically permissible for the husband to take on the burden of the disclosure without having the required special skills? In a healthcare context, it is known that breaking news to patients has been a subject of professional concern for many years, with interest growing alongside a culture of increasing medical disclosure of diagnosis and prognosis. Research demonstrates that breaking serious news can be an emotionally burdensome and intrinsically difficult task for healthcare professionals (2). Therefore, in this case, would it be best left to healthcare professionals, or would it be far better if the responsibility were shared? This, therefore, compels healthcare professionals to elucidate if the husband understands the nature of breaking the news and whether such a difficult position is best filled by healthcare professionals. Although the discussion of who has ‘jurisdiction’ over disclosing the tragic news is salient to some degree, perhaps it would be far more considerate to support the husband before and after breaking the news. While the use of protocols such as setting, perception, invitation, knowledge, empathy and strategy (SPIKES) (3) works on the assumption that breaking news is a clinical task, the protocol may be helpful in some ways to guide the husband. It may be most significant to guide the husband regarding when and how to break the news timely and sensitively. Receiving timely and appropriate information about a child’s death is an integral part of the grieving process for parents. Another potential dilemma the husband and the healthcare professionals face is how much information should be shared with the patient (mother) and whether it is appropriate to do so. This question requires some weighing of benefits and harm. Simultaneously, caution needs to be applied when generalising results of ‘bad news’ research across cultures, as preferences regarding content and delivery may vary (4).

Secondly, having announced the news, what would be the healthcare professional’s ethical responsibility in advocating for and supporting the best interests of the bereaved parents? It seems that the greater moral duty for healthcare professionals is to help the parents go through grief. It is critical to reflect on what happens once the patient has received the news. Do we give them the space to grieve at their own pace or do we intervene? After a child’s death, parents describe a need for privacy. To what extent should privacy be maintained? Parents have defined privacy as a sense of seclusion from the sights, sounds and presence of others. Simple measures like eliminating alarms and minimising loud can all help foster privacy and respect. Parents require this compassionate environment to make decisions immediately after their child’s death and in the weeks and months that follow (5).

An act of kindness shown during the hour of need will help strengthen the healthcare professional — patient-family relationship, particularly when the patient cannot hear or process the information given when the news was told. Being prepared to answer those questions later can positively impact their experience and ongoing grieving processes. This warrants a duty of truth-telling. At the same time, healthcare professionals must ensure that patient’s best interests are primary, and such interests must reflect the preferences of the patient, not their assumptions. For example, most parents grieving the loss of a child have benefited from personal words and acts of condolence from people who care for them (6).

However, it is crucial to recognise that in Eastern culture, where family is the core, they may be the best resource of support. Healthcare professionals must be careful in assuming and taking ownership of the support mechanism. Further assessment is needed since there is a suggestion that friends and family may not be effective at providing support and that cultural proscriptions to suppress mourning may impede healing for some (7). This may mean that a bereaved parent’s natural support system may often be insufficient to meet their needs, and societal expectations hasten and misunderstand grieving parents. In this case, healthcare professionals involved in such cases must develop appropriate knowledge, skills and understanding to support bereaved parents within the hospital and community settings.

Meanwhile, a bereaved person may become ‘stuck’ in the grieving process (8). In this case, the question is, how far can we go in supporting the parents? Are there and should there be limits in helping these parents? This is particularly relevant when cultural expectations and gender role socialisation play an essential role in shaping grief experiences. For example, women are expected to be emotional in grief, but men have been programmed not to show feelings and ‘act strong’ for the sake of their grieving spouse (9).

Considering the vulnerability of the bereaved parents, healthcare professionals have a greater moral duty toward a more sensitive approach. Healthcare professionals must not forget or dismiss the fact that the patient is also a survivor in this case. For her, communication with family after the death of a child is never going to be easy. Previous studies have suggested that bereaved parents following traumatic events such as road traffic accidents have suffered emotional consequences, including depression, grief, and posttraumatic stress disorder, that often persist years after their child’s death (10, 11). This includes the potential weight of parental guilt: as found in numerous studies whereby many parents hold themselves responsible (12). This calls for greater sensitivity and additional ethical responsibilities for everyone, including healthcare professionals, to prepare the patient as the news unfolds. Most importantly, every parent grieves differently, so healthcare professionals must reflect on highly individualised bereavement care. At the same time, there may also be a need to address societal expectations, something which they may or may not be ready to face in the community.

In short, I wish to reiterate here that further deliberation by healthcare professionals is needed to reflect the ethical quandary in the context of breaking the news and ethical responsibility when supporting bereaved parents. After all, there is no one-size-fits-all approach to how the parents are going to cope with what has happened. The case nonetheless reflects greater vulnerability for grieving parents in accepting what has happened. Healthcare professionals must also be sensitive to their ethical responsibility to promote the best interests of bereaved parents.
